# Poly[[(acetonitrile)­lithium(I)]-μ_3_-tetra­fluoridoborato]

**DOI:** 10.1107/S1600536811012141

**Published:** 2011-04-07

**Authors:** Daniel M. Seo, Paul D. Boyle, Wesley A. Henderson

**Affiliations:** a Department of Chemical & Biomolecular Engineering, North Carolina State University, Raleigh, NC 27695, USA; b Department of Chemistry, North Carolina State University, Raleigh, NC 27695, USA

## Abstract

The structure of the title compound, [Li(BF_4_)(CH_3_CN)]_*n*_, consists of a layered arrangement parallel to (100) in which the Li^+^ cations are coordinated by three F atoms from three tetra­fluoridoborate (BF_4_^−^) anions and an N atom from an acetonitrile mol­ecule. The BF_4_^−^ anion is coordinated to three different Li^+^ cations though three F atoms. The structure can be described as being built from vertex-shared BF_4_ and LiF_3_(NCCH_3_) tetra­hedra. These tetra­hedra reside around a crystallographic inversion center and form 8-membered rings.

## Related literature

For related compounds containing Li(BF_4_), see: Andreev *et al.* (2005 ▶); Henderson *et al.* (2003*a* ▶,*b* ▶); Ramirez *et al.* (2003 ▶); Francisco & Williams (1990 ▶). For the structures of related Li salts with CH_3_CN , see: Klapötke *et al.* (2006 ▶); Brooks *et al.* (2002 ▶); Yokota *et al.* (1999 ▶); Raston *et al.* (1989 ▶).
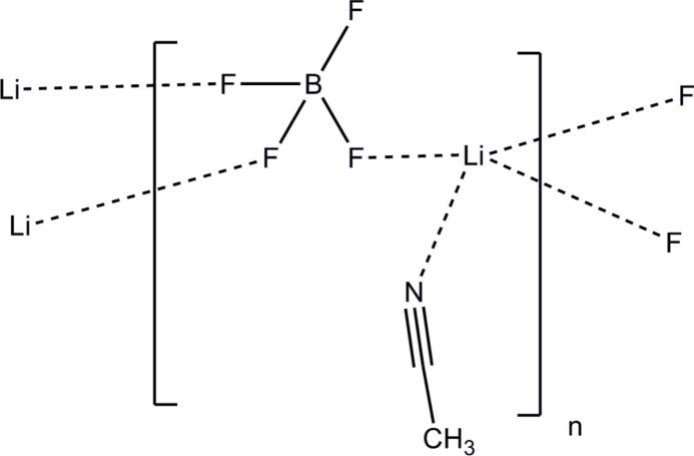


## Experimental

### 

#### Crystal data

[Li(BF_4_)(C_2_H_3_N)]
*M* *_r_* = 134.80
Monoclinic, 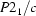

*a* = 7.8248 (6) Å
*b* = 8.8187 (7) Å
*c* = 8.2932 (6) Å
β = 95.5708 (18)°
*V* = 569.57 (8) Å^3^
*Z* = 4
Mo *K*α radiation
μ = 0.18 mm^−1^
*T* = 110 K
0.34 × 0.26 × 0.16 mm

#### Data collection

Bruker–Nonius Kappa X8 APEXII diffractometer
Absorption correction: multi-scan (*SADABS*; Bruker, 2009 ▶) *T*_min_ = 0.941, *T*_max_ = 0.971
13920 measured reflections
2650 independent reflections
2001 reflections with *I* > 2σ(*I*)
*R*_int_ = 0.037

#### Refinement

*R*[*F* ^2^ > 2σ(*F* ^2^)] = 0.045
*wR*(*F* ^2^) = 0.118
*S* = 1.05
2650 reflections
94 parameters
All H-atom parameters refined
Δρ_max_ = 0.43 e Å^−3^
Δρ_min_ = −0.18 e Å^−3^

### 

Data collection: *APEX2* (Bruker, 2009 ▶); cell refinement: *SAINT* (Bruker, 2009 ▶); data reduction: *SAINT*; program(s) used to solve structure: *SHELXS97* (Sheldrick, 2008 ▶); program(s) used to refine structure: *SHELXTL* (Sheldrick, 2008 ▶); molecular graphics: *ORTEP-3* (Farrugia, 1997 ▶); software used to prepare material for publication: *cif2tables.py* (Boyle, 2008 ▶).

## Supplementary Material

Crystal structure: contains datablocks I, global. DOI: 10.1107/S1600536811012141/fj2410sup1.cif

Structure factors: contains datablocks I. DOI: 10.1107/S1600536811012141/fj2410Isup2.hkl

Additional supplementary materials:  crystallographic information; 3D view; checkCIF report

## Figures and Tables

**Fig. 1.**
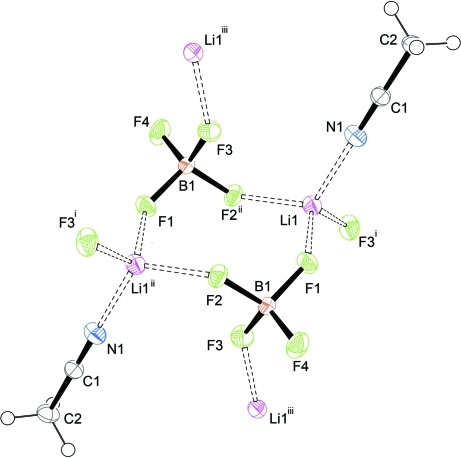
Molecular structure of the title compound. The thermal ellipsoids are shown at a 50% probability level. (Symmetric codes: (i) x, -y + 3/2, z - 1/2; (ii) -x, -y + 1, -z + 2; (iii) x, -y + 3/2, z + 1/2.)

**Fig. 2.**
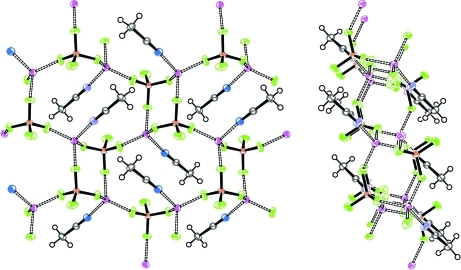
Packing diagram for the title compound.

## supplementary crystallographic information

### Comment

In this structure, atoms F1 and F2 are endocyclic linking the boron atom to the lithium atom while F3 and F4 are exocyclic. Neighboring rings are linked through a Li1—F3 bond to form an infinite two dimensional network which orients parallel to (1 0 0). The interface between the two dimensional networks is occupied by the aliphatic ends of the acetonitrile molecules and the F4 atoms and is largely at van der Waal contact distances. There is, however, a close intermolecular contact of 3.1601 (11) Å between the nitrile carbon atom, C1, and F4 (1 - *x*, 1 - *y*, 2 - *z*).

Solvate structures provide significant insight into the species which may exist in electrolytes solutions. Solvates based upon acetonitrile and lithium salts are particularly noteworthy as dinitrile solvents gain increasing interest as high-voltage solvents for lithium battery electrolytes. The phase diagram for (CH_3_CN)_n_—LiBF_4_ mixtures indicates that at least three different solvates may form with 4/1 (*T*_m_ = -12°C), 2/1 (*T*_m_ = 25°C) and 1/1 (*T*_m_ = 63°C) AN/Li compositions. The 4/1 solvate may resemble that for LiClO_4_ in which the Li^+^ cations are fully solvated by four acetonitrile molecules and the anions are uncoordinated. The 2/1 solvate, in turn, may resemble that for LiBr in which the Li^+^ cations are solvated by two acetonitrile molecules and two anions to form aggregated dimer solvates. The 1/1 solvate structure is reported here.

### Experimental

LiBF_4_ (99.998%) was purchased from Sigma-Aldrich and used as-received. Anhydrous acetonitrile (Sigma Aldrich, 99.8%) was used as-received. In a Vacuum Atmospheres inert atmosphere (N_2_) glove box (< 5 p.p.m. H_2_O), LiBF_4_ (1 mmol) and acetonitrile (1.5 mmol) were sealed in a vial and the mixture heated on a hot plate to form a homogeneous solution. Upon standing at ambient temperature, colorless plate single crystals suitable for analysis formed.

### Refinement

The structure was solved by direct methods using the XS program. All non-hydrogen atoms were obtained from the initial solution. The hydrogen atoms were introduced at idealized positions and were allowed to refine isotropically. The structural model was fit to the data using full matrix least-squares based on *F^2^*. The calculated structure factors included corrections for anomalous dispersion from the usual tabulation. The structure was refined using the XL program from *SHELXTL*, and graphic plots were produced using the *ORTEP-3* program.

### Crystal data

**Table:**

[Li(BF_4_)(C_2_H_3_N)]	*F*(000) = 264
*M**_r_* = 134.80	*D*_x_ = 1.572 Mg m^−^^3^
Monoclinic, *P*2_1_/*c*	Mo *K*α radiation, λ = 0.71073 Å
Hall symbol: -P 2ybc	Cell parameters from 2729 reflections
*a* = 7.8248 (6) Å	θ = 2.6–29.5°
*b* = 8.8187 (7) Å	µ = 0.18 mm^−^^1^
*c* = 8.2932 (6) Å	*T* = 110 K
β = 95.5708 (18)°	Prism, colourless
*V* = 569.57 (8) Å^3^	0.34 × 0.26 × 0.16 mm
*Z* = 4	

### Data collection

**Table:**

Bruker–Nonius Kappa X8 APEXII diffractometer	2650 independent reflections
Radiation source: fine-focus sealed tube	2001 reflections with *I* > 2σ(*I*)
graphite	*R*_int_ = 0.037
ω and φ scans	θ_max_ = 36.5°, θ_min_ = 2.6°
Absorption correction: multi-scan (*SADABS*; Bruker, 2009)	*h* = −13→12
*T*_min_ = 0.941, *T*_max_ = 0.971	*k* = −14→14
13920 measured reflections	*l* = −13→13

### Refinement

**Table:**

Refinement on *F*^2^	Primary atom site location: structure-invariant direct methods
Least-squares matrix: full	Secondary atom site location: difference Fourier map
*R*[*F*^2^ > 2σ(*F*^2^)] = 0.045	Hydrogen site location: inferred from neighbouring sites
*wR*(*F*^2^) = 0.118	All H-atom parameters refined
*S* = 1.05	*w* = 1/[σ^2^(*F*_o_^2^) + (0.0588*P*)^2^ + 0.0555*P*] where *P* = (*F*_o_^2^ + 2*F*_c_^2^)/3
2650 reflections	(Δ/σ)_max_ = 0.001
94 parameters	Δρ_max_ = 0.43 e Å^−^^3^
0 restraints	Δρ_min_ = −0.18 e Å^−^^3^

### Special details

**Table:**

Geometry. All e.s.d.'s (except the e.s.d. in the dihedral angle between two l.s. planes) are estimated using the full covariance matrix. The cell e.s.d.'s are taken into account individually in the estimation of e.s.d.'s in distances, angles and torsion angles; correlations between e.s.d.'s in cell parameters are only used when they are defined by crystal symmetry. An approximate (isotropic) treatment of cell e.s.d.'s is used for estimating e.s.d.'s involving l.s. planes.
Refinement. Refinement of *F*^2^ against ALL reflections. The weighted *R*-factor *wR* and goodness of fit *S* are based on *F*^2^, conventional *R*-factors *R* are based on *F*, with *F* set to zero for negative *F*^2^. The threshold expression of *F*^2^ > σ(*F*^2^) is used only for calculating *R*-factors(gt) *etc*. and is not relevant to the choice of reflections for refinement. *R*-factors based on *F*^2^ are statistically about twice as large as those based on *F*, and *R*- factors based on ALL data will be even larger.

### Fractional atomic coordinates and isotropic or equivalent isotropic displacement parameters (Å^2^)

**Table:**

	*x*	*y*	*z*	*U*_iso_*/*U*_eq_	
Li1	0.0962 (2)	0.60760 (19)	0.7753 (2)	0.0207 (3)	
N1	0.21544 (11)	0.47042 (10)	0.62923 (10)	0.02471 (18)	
C1	0.28947 (11)	0.38756 (11)	0.55611 (11)	0.01953 (17)	
C2	0.38544 (13)	0.28199 (12)	0.46485 (13)	0.02381 (19)	
H2A	0.447 (3)	0.336 (2)	0.392 (2)	0.058 (5)*	
H2B	0.309 (2)	0.211 (2)	0.408 (2)	0.049 (5)*	
H2C	0.461 (3)	0.223 (2)	0.533 (2)	0.059 (5)*	
B1	0.19662 (13)	0.58984 (12)	1.14638 (12)	0.01831 (18)	
F1	0.21991 (8)	0.59642 (7)	0.98110 (7)	0.02477 (14)	
F2	0.12648 (7)	0.44806 (6)	1.17989 (8)	0.02285 (14)	
F3	0.07681 (9)	0.70149 (7)	1.17969 (9)	0.03087 (16)	
F4	0.34930 (8)	0.61145 (9)	1.23724 (8)	0.03467 (19)	

### Atomic displacement parameters (Å^2^)

**Table:**

	*U*^11^	*U*^22^	*U*^33^	*U*^12^	*U*^13^	*U*^23^
Li1	0.0227 (7)	0.0198 (7)	0.0202 (8)	0.0011 (6)	0.0048 (6)	0.0011 (6)
N1	0.0279 (4)	0.0250 (4)	0.0220 (4)	0.0037 (3)	0.0060 (3)	0.0003 (3)
C1	0.0205 (4)	0.0204 (4)	0.0176 (4)	−0.0002 (3)	0.0016 (3)	0.0005 (3)
C2	0.0252 (4)	0.0239 (4)	0.0227 (4)	0.0038 (3)	0.0045 (3)	−0.0062 (4)
B1	0.0195 (4)	0.0181 (4)	0.0175 (4)	−0.0047 (3)	0.0030 (3)	−0.0010 (3)
F1	0.0258 (3)	0.0324 (3)	0.0164 (3)	−0.0019 (2)	0.0035 (2)	0.0009 (2)
F2	0.0219 (3)	0.0168 (3)	0.0304 (3)	−0.00237 (19)	0.0056 (2)	0.0023 (2)
F3	0.0368 (3)	0.0182 (3)	0.0399 (4)	−0.0010 (2)	0.0154 (3)	−0.0065 (2)
F4	0.0272 (3)	0.0515 (4)	0.0238 (3)	−0.0175 (3)	−0.0052 (2)	0.0047 (3)

### Geometric parameters (Å, °)

**Table:**

Li1—F3^i^	1.8609 (18)	C2—H2B	0.956 (18)
Li1—F1	1.8810 (18)	C2—H2C	0.935 (19)
Li1—F2^ii^	1.8820 (18)	B1—F4	1.3626 (11)
Li1—N1	2.0051 (19)	B1—F1	1.4013 (12)
N1—C1	1.1426 (12)	B1—F2	1.4041 (11)
C1—C2	1.4539 (13)	B1—F3	1.4053 (12)
C2—H2A	0.94 (2)		
			
F3^i^—Li1—F1	116.59 (9)	H2A—C2—H2C	109.8 (16)
F3^i^—Li1—F2^ii^	106.33 (9)	H2B—C2—H2C	105.1 (16)
F1—Li1—F2^ii^	102.23 (8)	F4—B1—F1	110.18 (8)
F3^i^—Li1—N1	108.18 (9)	F4—B1—F2	110.71 (8)
F1—Li1—N1	106.74 (8)	F1—B1—F2	108.72 (8)
F2^ii^—Li1—N1	117.12 (9)	F4—B1—F3	111.05 (8)
C1—N1—Li1	174.92 (10)	F1—B1—F3	108.41 (8)
N1—C1—C2	179.24 (10)	F2—B1—F3	107.69 (7)
C1—C2—H2A	109.4 (12)	B1—F1—Li1	141.75 (8)
C1—C2—H2B	110.4 (11)	B1—F2—Li1^ii^	131.19 (7)
H2A—C2—H2B	110.3 (16)	B1—F3—Li1^iii^	133.56 (8)
C1—C2—H2C	111.7 (11)		
			
F4—B1—F1—Li1	−168.69 (11)	F4—B1—F2—Li1^ii^	132.27 (10)
F2—B1—F1—Li1	69.82 (15)	F1—B1—F2—Li1^ii^	−106.58 (11)
F3—B1—F1—Li1	−46.99 (15)	F3—B1—F2—Li1^ii^	10.69 (13)
F3^i^—Li1—F1—B1	99.32 (14)	F4—B1—F3—Li1^iii^	18.68 (14)
F2^ii^—Li1—F1—B1	−16.17 (16)	F1—B1—F3—Li1^iii^	−102.49 (12)
N1—Li1—F1—B1	−139.69 (11)	F2—B1—F3—Li1^iii^	140.04 (10)

Symmetry codes: (i) *x*, −*y*+3/2, *z*−1/2; (ii) −*x*, −*y*+1, −*z*+2; (iii) *x*, −*y*+3/2, *z*+1/2.

## References

[bb1] Andreev, Y. G., Seneviratne, V., Khan, M., Henderson, W. A., Frech, R. E. & Bruce, P. G. (2005). *Chem. Mater.* **17**, 767–772.

[bb2] Boyle, P. D. (2008). http://www.xray.ncsu.edu/PyCIFUtils/

[bb3] Brooks, N. R., Henderson, W. A. & Smyrl, W. H. (2002). *Acta Cryst.* E**58**, m176–m177. 10.1107/s0108270102015135 12359926

[bb4] Bruker (2009). *SAINT* and *SADABS* Bruker AXS Inc., Madison, Wisconsin, USA.

[bb5] Farrugia, L. J. (1997). *J. Appl. Cryst.* **30**, 565.

[bb6] Francisco, J. S. & Williams, I. H. (1990). *J. Phys. Chem.* **94**, 8522–8529.

[bb7] Henderson, W. A., Brooks, N. R., Brennessel, W. W. & Young, V. G. Jr (2003*a*). *Chem. Mater.* **15**, 4679–4684.

[bb8] Henderson, W. A., Brooks, N. R., Brennessel, W. W. & Young, V. G. Jr (2003*b*). *Chem. Mater.* **15**, 4685–4690.

[bb9] Klapötke, T. M., Krumm, B., Mayer, P., Scherr, M. & Schwab, I. (2006). *Acta Cryst.* E**62**, m2666–m2667.

[bb10] Ramirez, A., Lobkovosky, E. & Collum, D. B. (2003). *J. Am. Chem. Soc.* **125**, 15376–15387. 10.1021/ja030322d 14664582

[bb11] Raston, C. L., Whitaker, C. R. & White, A. H. (1989). *Aust. J. Chem.* **42**, 201–207.

[bb12] Sheldrick, G. M. (2008). *Acta Cryst.* A**64**, 112–122. 10.1107/S0108767307043930 18156677

[bb13] Yokota, Y., Young, V. G. & Verkade, J. G. (1999). *Acta Cryst.* C**55**, 196–198.

